# Socioeconomic Status and Physical Activity in Chinese Adults: A Report from a Community-Based Survey in Jiaxing, China

**DOI:** 10.1371/journal.pone.0132918

**Published:** 2015-07-15

**Authors:** Mingling Chen, Yikang Wu, Hiroto Narimatsu, Xueqing Li, Chunmei Wang, Jianyong Luo, Genming Zhao, Zhongwen Chen, Wanghong Xu

**Affiliations:** 1 Department of Epidemiology, School of Public Health and Key Laboratory of Public Health Safety, Fudan University, Shanghai, China; 2 Jiaxing Center for Disease Control and Prevention, Jiaxing, China; 3 Department of Public Health, Yamagata University Graduate School of Medicine, 2-2-2, Iida-nishi, Yamagata, Japan; 4 Tongxiang Center for Disease Control and Prevention, Tongxiang, China; Zhejiang University, CHINA

## Abstract

**Objectives:**

This study examines the associations of socioeconomic status (SES) with intensity of different types of physical activity (PA) in Chinese adults, aimed at outlining and projecting socioeconomic disparities in PA among the population undergoing a rapid nutrition transition.

**Methods:**

A community-based survey was conducted among 3,567 residents aged 30–65 years old in Jiaxing, China, in 2010. SES and PA were assessed by a structured questionnaire. SES was assessed as socioeconomic index (SEI) score based on self-reported educational attainment, household income and occupation. Metabolic equivalents (METs) were calculated for each subject to quantify the total amount of PA from occupation, exercise, transportation and housework.

**Results:**

Intensity of overall PA in this population was 165 MET-hours/week, in which energy expenditure in occupational PA accounted for 82%. Both types and intensity of PA were significantly different by SES: middle SES groups had higher intensity of occupational activities; lower SES subjects engaged in more household work; whereas higher SES subjects were more likely to exercise, more active during commuting and had longer sedentary time. All the three components of SES, education attainment, income and occupation, contributed to socioeconomic disparities in PA in this population.

**Conclusions:**

Our results suggest an overall insufficiency and socioeconomic inequalities in PA among Chinese adults in Jiaxing, a typical city experiencing a rapid urbanization in China. There is an urgent need to promote leisure-time activities in this population.

## Introduction

Physical inactivity, identified as the fourth leading cause of mortality [1], has been becoming a major health concern globally. Of the four modifiable risk factors for non-communicable chronic diseases (NCDs), namely, tobacco use, alcohol consumption, unhealthy diet, and physical inactivity, lack of physical activity (PA) ranks the first in prevalence across populations [2]. Since the landmark paper published in *The Lancet* reported the association of PA with the risk of coronary heart-disease [3], the rapidly accumulating literature has suggested that engaging in regular PA may reduce the risk of various adverse health conditions, including coronary heart disease, hypertension, stroke, type 2 diabetes, colon cancer, breast cancer, depression and other NCDs [4,5,6,7]. World Health Organization (WHO) recommends at least 150 minutes moderate-intensity PA or 75 minutes vigorous-intensity PA per week for adults [8].

Many factors may influence the engagement of PA. The role of socioeconomic status (SES) in PA behaviors has attracted much attention in recent years. SES is an economic and sociological combined measure of a person's position within a hierarchical social structure, based on income, education, and occupation [9]. Studies conducted in developed countries consistently observed a higher level of leisure-time PA in upper SES adults, and a higher intensity of occupational activity among lower SES groups [10,11,12]. Of the three components of SES, education has been most strongly associated with PA intensity in these populations [13,14,15].

As the vast majority of this research focused on developed countries, only several studies have examined the patterns and correlates of PA among Chinese adults [16,17,18]. Unlike in developed countries where leisure-time and occupational activities were the main types of PA [10], in China occupational and household labor have turned out to be the largest contributors to overall PA [19,20]. Due to the rapid urbanization and the use of modern labor-saving technologies at the workplace and household, however, the two main types of PA have been decreasing significantly during the past decades [19,20]. PA patterns have been associated with the components of SES such as education, occupation and income level, respectively, in Chinese adults [16,17,18]. Comprehensively measured SES and PA data are needed to better understand the health inequality existing in this population which may differ from that in their western counterparts.

In this study, we used the comprehensive data from a community-based survey conducted in Jiaxing, Zhejiang Province of China, to examine the associations of SES and its components with different types of PA. Located at Yangtze River Delta, one of the most economically developed regions of China, Jiaxing has witnessed a dramatic growth of urbanization from 11.8% in 1980 to 53.3% in 2010, which is twice as fast as the world average growth at the same period [21]. According to the Sixth National Population Census, about 1.22 million residents of Jiaxing city were from the rural areas by the end of 2010, accounting for 27.2% of the residents. The results from this typical population who is experiencing a rapid urbanization and nutrition transition may help to outline socioeconomic inequalities in PA in China, and help to develop effective health policies and strategies to prevent NCDs.

## Materials and Methods

### Study design and subjects

We used the baseline data from a longitudinal study to monitor changes in lifestyles and health outcomes in adults residing in Jiaxing, China. The baseline survey was conducted from April to May 2010. A total of 4,000 eligible local residents from the communities of Jiaxing city were recruited by using the multi-stage random sampling method. First, two streets and two towns were randomly selected from a total of 29 streets and 44 towns in Jiaxing city. And then 1,000 eligible residents were randomly selected from each street or town according to the predetermined sex and age distribution. Inclusion criteria included permanent residents of Jiaxing at the age of 30–65 years old, and willingness to complete at least two surveys over one year. Those physically or mentally disabled were excluded from the study.

Physicians from local community healthcare centers were trained as interviewers. A structured in-person interview was conducted to collect information on demographic factors (gender, birthdate, educational level, occupation, income per capita), lifestyle behaviors (physical activities, cigarette smoking and alcohol drinking), diagnosis of type 2 diabetes, hypertension, coronary heart disease, stroke, myocardial infarction, chronic obstructive pulmonary disease, asthma and cancer, and family history of type 2 diabetes, hypertension, coronary heart disease, stroke and cancer. Education level was categorized as illiterate or semiliterate, elementary school (5 or 6 years), middle school (7~9 years), high school (10~12 years) and college or above (>12 years). Occupation was classified as administration staff, professional, clerk, service personnel, manual worker and unemployed based on self-reported job descriptions. Annual income per capita was defined as total annual household income divided by the number of family members. Cigarette smoking was defined as having smoked at least 100 cigarettes in one’s lifetime. Alcohol drinking was defined as consumption of any alcohol beverage at least once a week during the last year.

At the interview, body weight, standing height and waist circumference (WC) were measured for each participant according to a standard protocol [22]. Body weight and standing height were measured to the nearest 0.1 kg and 0.1 cm respectively, with the subject standing barefoot in light clothes. Waist circumference was measured to the nearest 0.1 cm at the approximate midpoint between the lower margin of the last palpable rib and the top of the iliac crest. Body mass index (BMI) was then calculated as body weight in kilograms divided by standing height in meters squared.

Of the 4,000 eligible subjects approached at baseline, 3,973 (99.3%) participated in the survey and 27 (0.7%) declined or were absent during the study period. We further excluded 341 subjects without information on occupation and 65 with incomplete information on PA. Finally, 3,567 subjects were included in our analysis. The study was granted approval by the Institutional Review Committee of Jiaxing Center for Disease Control and Prevention. All participants provided their written informed consents.

### Measurement of physical activities

The physical activity questionnaire was derived from the China Health and Nutrition Survey (CHNS), a nationwide study monitoring extensive individual-level information on demography, health and lifestyles [23]. In the questionnaire, five types of PA were included: occupational activities, exercise, transportation activities, housework and sedentary behaviors. For each type of PA, frequency, duration and intensity were measured to calculate the energy expenditure. All measures were then presented in terms of metabolic equivalent (MET)-hours/week or hours/week using standard methods [24]. Overall intensity of PA was obtained by summing up energy consumption in occupational, exercise, travel and domestic domains.

Occupational activities were classified into four levels (very low, low, moderate, and vigorous levels) based on job descriptions and the time spent in sitting, standing, walking and lifting heavy loads during an average work day. Each level was assigned a MET value according to Compendium of Physical Activities [25]: 1.5 METs for teacher, secretary, office staff, etc.; 2.5 METs for tailor, driver, seller, craftsman, etc.; 4 METs for carpenter, cleaner, bricklayer, gardener, etc.; and 8 METs for steel worker, heavy loads carrier, etc. Then these MET values were multiplied by the hours spent in each occupation per week during the last year to calculate the energy expenditure. For participants from the suburbs, their part-time farm work was also included in occupational activities.

Exercise was categorized into two intensity levels. Vigorous exercise referred to activities that cause large increases in breathing or heart rate like running, playing basketball or tennis, while moderate exercises were defined as those causing a small increase in breathing or heart rate such as dancing, yoga, tai chi, etc. Transportation activities included bicycling and walking during commuting, exercise or shopping that last at least 10 minutes in a typical week. Respondents were asked about their participation in exercise, transportation mode, and the average time spent per week during the past year. As questions on exercise and transportation activities were derived from Global Physical Activity Questionnaire (GPAQ), we applied the recommended METs using WHO guideline: 4 METs for moderate exercise, 8 METs for vigorous exercise, 4 METs for both cycling and walking [26].

All subjects also reported their average hours/week spent on housework and sedentary behaviors in the previous year. Housework included a number of key household tasks such as cooking or preparing food, washing dishes, doing laundry, cleaning the house and child care that last at least 10 minutes each time. An average value of 3 METs was assigned to calculate the energy expenditure in household activities according to Compendium of Physical Activities [25]. Sedentary time included time spent on TV watching, computer using, video game playing, and reading at home, in car or with friends, but not during working.

### Assessment of socioeconomic status

Socioeconomic index (SEI) score was calculated to measure SES based on educational attainment, occupation and income per capita. In this study, we used Li’s scale for Chinese urban residents (version 2010) [27] as a standard scale. Li’s scale, commonly used in the social science research in China [28,29,30], was modified based on the scale first proposed by Duncan [31], and is updated every year because income per capita, one component of SES, changes with Consumer Price Index Numbers for Industrial Workers (CPI-IW) [9]. Educational attainment, occupation and income per capita were classified to assign scores and summarized as a comprehensive SEI score, as described in **S1 Table**. Then based on SEI scores, all subjects were classified as Upper (≥ 12 scores), Upper middle (9~11 scores), Lower middle (6~8 scores) and Lower SES class (≤ 5 scores).

### Statistical analysis

Sex differences in demographic and socioeconomic characteristics were evaluated by using χ^2^ tests (categorical variables) and *t*-tests or Wilcoxon rank sum tests (continuous variables). Demographic, clinical and lifestyle factors across SES levels were compared using χ^2^ tests (for categorical variables) or analysis of variance (ANOVA) (for continuous variables). Intensity of PA in METs were square root transformed to approximate normal distribution and then used to calculate lsmean and 95% confidence interval (CI) accounting for age and sex by SES levels. Trends in participation rates of PA by SES were evaluated by Cochran-Mantel-Haenszel χ^2^ tests, while those in intensity of PA by SES were evaluated by ANOVA. Potential dose-response relationship of SEI score with PA intensity (METs) was evaluated using restricted cubic splines (RCS). Beta coefficients and 95% CIs for each SES component related to PA intensity (METs) were derived from generalized linear modeling (GLM). Tests for linear trend were performed by entering the categorical variables as continuous parameters in the adjusted models. All analyses were performed using SAS version 9.2 for windows (SAS Institute, Cary, North Carolina), and all tests of statistical significance were based on two-tailed probability.

## Results

Of 3,567 participants with an average age of 45.5 years, 1,757 (49.3%) were men and 1,810 (50.7%) were women. As shown in **Table 1**, no significant difference was observed in annual income per capita between men and women. However, men were older, had higher educational level and were more likely to engage in manual, administrative and professional work compared with women, and had a higher average SEI score than women (*P*<0.0001).

**Table 1:** Socioeconomic characteristics of study participants, the community-based survey in Jiaxing, China, 2010.

	All subjects	Men	Women	
	(N = 3,567)	(N = 1,757)	(N = 1,810)	*P* value ^b^
Age (years, mean±SD)	45.5±6.7	46.1±6.7	44.9±6.5	*<0*.*0001*
Education (%)				*<0*.*0001*
Illiterate or semiliterate	10.1	5.0	15.0	
Elementary school	28.3	26.2	30.3	
Middle school	44.8	49.4	40.4	
High school	13.1	15.0	11.3	
College or above	3.7	4.4	3.0	
Occupation (%)				*<0*.*0001*
Administration staff	2.3	3.4	1.3	
Professional	5.9	7.7	4.1	
Clerk	4.8	4.2	5.4	
Service personnel	19.9	18.7	21.0	
Manual worker	58.3	60.3	56.4	
Unemployed	8.8	5.7	11.8	
Annual income per capita (USD) ^a^	1967 (1639, 3279)	1967 (1639, 3279)	1967 (1639, 3279)	*0*.*2335*
Socioeconomic index score ^a^	7 (6, 9)	8 (7, 9)	7 (6, 9)	*<0*.*0001*

Abbreviations: SD: standard deviation; USD: United States dollar.

^a^ Presented as median (25^th^, 75^th^ percentile).

^b^ *P* for χ^2^ tests (categorical variables) and *t*-tests or Wilcoxon rank sum tests (continuous variables).

**Table 2** presents demographic, clinical and lifestyle factors of participants by SES levels. The participants with lower SES were older. After adjusting for age and sex, higher BMI and WC were observed among men with higher SES and women in lower classes, respectively. Regarding lifestyles, men with lower SES were more likely to smoke, while women with higher SES tended to consume alcohol. No significant difference was observed in diagnosis of NCDs by SES, but family history of NCDs was more prevalent in the Upper-middle class.

**Table 2:** Demographic, clinical and lifestyle factors of study participants by socioeconomic status, the community-based survey in Jiaxing, China, 2010.

	Socioeconomic status				
	Lower class	Lower middle class	Upper middle class	Upper class	*P for trend* ^d^
	(n = 391)	(n = 2100)	(n = 771)	(n = 305)	
Age (years, mean±SD)	50.7±6.7	45.3±6.5	44.2±6.1	43.5±6.2	*<0*.*0001*
Sex (%)					*<0*.*0001*
Men	31.5	49.6	53.7	58.4	
Women	68.5	50.4	46.3	41.6	
Body mass index (kg/m^2^) ^a^					
Men	23.7 (23.2, 24.3)	23.9 (23.7, 24.1)	24.3 (24.0, 24.6)	24.3 (23.8, 24.7)	*0*.*0104*
Women	24.6 (24.2, 25.0)	23.8 (23.6, 24.0)	23.0 (22.7, 23.3)	23.1 (22.5, 23.6)	*<0*.*0001*
Waist circumference (cm) ^a^					
Men	83.3 (81.6, 84.9)	84.1 (83.6, 84.7)	85.3 (84.4, 86.2)	84.9 (83.5, 86.2)	*0*.*0219*
Women	82.2 (81.2, 83.3)	79.9 (79.4, 80.4)	76.8 (75.9, 77.7)	76.4 (74.9, 77.8)	*<0*.*0001*
Diagnosis of NCDs (%) ^b^ *	31.6	19.9	17.3	20.7	*0*.*4954*
Selected NCDs in 1^st^ degree relatives(%) ^c^ *	59.0	61.8	65.4	62.7	*0*.*0089*
Cigarette smoking (%) *					
Men	82.6	78.6	72.7	66.1	*<0*.*0001*
Women	3.0	1.1	1.4	0.8	*0*.*2482*
Alcohol drinking (%) *					
Men	47.1	41.6	46.0	44.6	*0*.*4129*
Women	0.8	3.3	8.5	5.6	*<0*.*0001*

Abbreviations: NCD: non-communicable chronic disease.

* Missing values excluded from analysis (14 for prevalent NCDs, 50 for family history, and 77 for cigarette and alcohol use).

^a^ Presented as Ls-means and 95% confidence intervals adjusted for age.

^b^ Including type 2 diabetes, hypertension, coronary heart disease, stroke, myocardial infarction, chronic obstructive pulmonary disease, asthma and cancer.

^c^ Including type 2 diabetes, hypertension, coronary heart disease, stroke and cancer.

^d^ *P* for ANOVA tests (continuous variables) or Cochran-Mantel-Haenszel χ^2^ tests (categorical variables), all *P* values adjusted for age and sex.

In this population, energy expenditure in occupational activities accounted for 82% of total energy expenditure in PA. As presented in **Table 3**, both PA type and intensity differed significantly by SES. Subjects with higher SES were more likely to engage in occupational activities and exercise but less likely to do housework (all *P* for trend <0.01). However, unlike the energy expenditure in housework decreasing along with increasing SES in this population (*P* for trend <0.0001), the energy expenditure in exercise was five times more in the two upper SES groups than in the two lower ones, and that in occupational activities was lower in the two extreme groups than in the middle classes. The participants in the Upper and Lower classes were also more likely to have transportation activities. Specifically, the energy expenditure in walking increased with increasing SES level (*P* for trend <0.0001), but that in cycling did not differ by SES (*P* for trend = 0.1897). As for sedentary time, a significantly positive association with SES was observed (*P* for trend <0.0001).

**Table 3:** Physical activity patterns of study participants by socioeconomic status, the community-based survey in Jiaxing, China, 2010.

Types of physical activity	Socioeconomic status				
	Lower class	Lower middle class	Upper middle class	Upper class	*P for trend* ^c^
	(n = 391)	(n = 2100)	(n = 771)	(n = 305)	
Occupational activity					
Participation (%)	72.1	91.6	96.2	99.3	*<0*.*0001*
Intensity (MET-hours/week/year) ^a^	84.5 (76.6,92.8)	131.3 (127.1,135.4)	108.6 (102.4,115.0)	84.7 (76.1,93.7)	*0*.*0002*
Exercise					
Participation (%)	7.4	8.1	18.2	23.6	*<0*.*0001*
Intensity (MET-hours/week) ^a^					
Moderate	0.04 (0.01,0.10)	0.05 (0.03,0.07)	0.21 (0.14,0.29)	0.29 (0.17,0.44)	*<0*.*0001*
Vigorous	0.04 (0.01,0.09)	0.03 (0.02,0.05)	0.10 (0.06,0.15)	0.19 (0.10,0.31)	*<0*.*0001*
Total	0.1 (0.0,0.2)	0.1 (0.1,0.2)	0.5 (0.4,0.7)	0.9 (0.6,1.2)	*<0*.*0001*
Transportation					
Participation (%)	40.4	27.7	36.2	47.9	*<0*.*0001*
Intensity (MET-hours/week) ^a^					
Cycling	0.2 (0.1,0.4)	0.2 (0.2,0.3)	0.2 (0.1,0.2)	0.2 (0.1,0.3)	*0*.*1897*
Walking	0.6 (0.3,0.9)	0.5 (0.4,0.6)	1.1 (0.9,1.4)	2.4 (1.8,3.1)	*<0*.*0001*
Total	1.4 (0.9,2.0)	1.2 (1.0,1.4)	2.0 (1.5,2.4)	3.6 (2.7,4.5)	*<0*.*0001*
Housework					
Participation (%)	89.0	82.5	79.1	72.1	*0*.*0023*
Intensity (MET-hours/week) ^a^	20.3 (18.4,22.3)	14.2 (13.5,14.9)	12.8 (11.8,13.9)	9.7 (8.3,11.3)	*<0*.*0001*
Overall intensity of PA (MET-hours/week) ^a^ ^,^ ^b^	140.3 (132.3,148.5)	167.4 (163.8,171.0)	141.1 (135.6,146.6)	114.4 (106.6,122.5)	*<0*.*0001*
Sedentary time (hours/week) ^a^	14.4 (13.1,15.7)	15.6 (15.1,16.1)	22.2 (21.3,23.1)	24.9 (23.5,26.3)	*<0*.*0001*

Abbreviations: MET: metabolic equivalent; PA: physical activity.

^a^ Presented as Ls-means and 95% confidence intervals adjusted for age and sex.

^b^ Including occupational activities, exercise, transportation activities and housework.

^c^ *P* for Cochran-Mantel-Haenszel χ^2^ tests (categorical variables) or ANOVA tests (continuous variables), all *P* values adjusted for age and sex.

**Fig 1:**
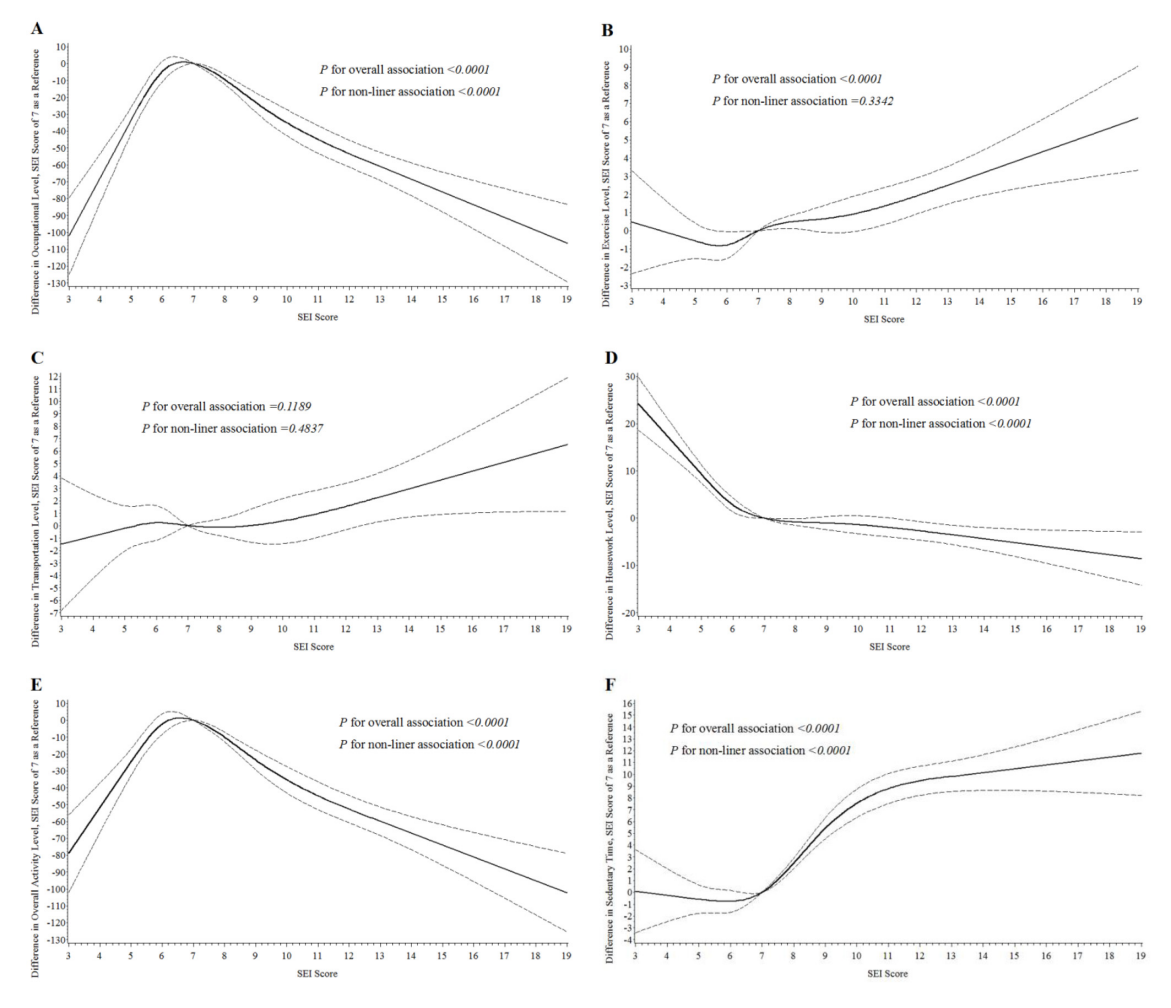
Associations of SEI score with overall and different types of PA level, the community-based survey in Jiaxing, China, 2010. Panel A-F: (A) Occupational activity, (B) Exercise, (C) Transportation activity, (D) Housework, (E) Overall activity, (F) Sedentary time. The solid lines indicate estimated mean change in PA level and the dotted lines represent 95% confidence intervals. The estimates were adjusted for age, sex, body mass index and waist circumference.

As indicated in **Fig 1**, SEI score was in a significant nonlinear association with sedentary time, energy expenditure in occupational activities and housework (all *P* values for nonlinear association <0.0001), and in a linear dose-response relationship with intensity of exercise (*P* for nonlinear association = 0.3342). No significant association was observed between SEI score and energy expenditure in transportation (*P* for overall association = 0.1189). As a result, METs from overall PA showed an upward trend when SEI score was less than 7, and then decreased continuously.

To better understand the contribution of each SES component to the engagement of PA, we further evaluated associations of education, occupation and income with intensity of each type of PA. As shown in **Table 4**, both higher educational attainment and higher income level were associated with lower intensity of occupational activities and housework independently, but just slightly or not related with higher intensity of exercise and transportation. In contrast with manual workers, both non-manual workers and unemployed subjects expended more energy in exercise and transportation activities but had lower intensity of occupational activities, particularly for those unemployed (β = -156.1, 95%CI:-164.2,-148.1). Correspondingly, high educational level, unemployed status and high income were linked to longer sedentary time.

**Table 4:** Associations of individual components of socioeconomic status with physical activity patterns, the community-based survey in Jiaxing, China, 2010.

SES components		Beta coefficient (95%CI) ^a^					
	No. of subjects	Occupational activities	Exercise	Transportation	Housework	Overall intensity of PA ^b^	Sedentary time
		(MET-hours/week/year)	(MET-hours/week)	(MET-hours/week)	(MET-hours/week)	(MET-hours/week)	(hours/week)
Educational level							
≤ Elementary school	1368	0.0 (Reference)	0.0 (Reference)	0.0 (Reference)	0.0 (Reference)	0.0 (Reference)	0.0 (Reference)
Middle school	1598	-10.6 (-15.9,-5.3)	1.0 (0.2,1.7)	-0.1 (-1.5,1.4)	-3.5 (-5.0,-1.9)	-13.1 (-18.7,-7.5)	3.7 (2.8,4.6)
High school	468	-38.6 (-46.1,-31.0)	0.8 (-0.3,1.9)	1.5 (-0.5,3.6)	-3.2 (-5.3,-1.0)	-39.3 (-47.3,-31.4)	10.1 (8.8,11.4)
≥ College	133	-62.6 (-75.4,-49.8)	1.0 (-0.9,2.8)	2.9 (-0.6,6.5)	-6.1 (-9.8,-2.4)	-64.8 (-78.3,-51.3)	9.6 (7.4,11.8)
*P for trend*		*<0*.*0001*	*0*.*0636*	*0*.*0809*	*<0*.*0001*	*<0*.*0001*	*<0*.*0001*
Occupation							
Manual worker	2079	0.0 (Reference)	0.0 (Reference)	0.0 (Reference)	0.0 (Reference)	0.0 (Reference)	0.0 (Reference)
Non-manual worker	1175	-29.8 (-35.0,-24.6)	2.9 (2.2,3.7)	1.8 (0.4,3.3)	-0.1 (-1.6,1.5)	-25.1 (-30.6,-19.6)	4.3 (3.4,5.2)
Unemployed ^c^	313	-156.1 (-164.2,-148.1)	6.1 (5.0,7.3)	2.4 (0.2,4.7)	11.5 (9.2,13.9)	-136.0 (-144.5,-127.5)	12.1 (10.7,13.4)
Annual income per capita (USD)							
≤ 984	332	0.0 (Reference)	0.0 (Reference)	0.0 (Reference)	0.0 (Reference)	0.0 (Reference)	0.0 (Reference)
985~1,967	1541	-12.6 (-20.5,-4.7)	1.0 (-0.2,2.1)	0.5 (-1.7,2.7)	-0.9 (-3.2,1.4)	-12.0 (-20.4,-3.6)	-0.6 (-1.9,0.8)
1,968~3,934	1176	-12.9 (-21.1,-4.7)	1.1 (-0.1,2.3)	-0.2 (-2.4,2.1)	-2.0 (-4.4,0.4)	-14.0 (-22.6,-5.3)	0.7 (-0.7,2.1)
≥ 3,935	518	-21.0 (-30.4,-11.5)	1.1 (-0.3,2.4)	0.1 (-2.5,2.7)	-3.6 (-6.4,-0.9)	-23.4 (-33.4,-13.5)	2.4 (0.8,4.1)
*P for trend*		*0*.*0002*	*0*.*1906*	*0*.*6679*	*0*.*0027*	*<0*.*0001*	*<0*.*0001*

Abbreviations: SES: socioeconomic status; CI: confidence interval.

^a^ Adjusted for age, sex, body mass index and waist circumference, with education, occupation and income mutually adjusted.

^b^ Including occupational activities, exercise, transportation activities and housework.

^c^ A total of 7 unemployed subjects sometimes engaged in farm work for their families.

The tests of collinearity showed that the tolerances (TOL) were 0.890, 0.936 and 0.932, respectively, for education, occupation and income, and the variance inflation factors (VIF) were 1.124, 1.068 and 1.073, respectively. The results indicated that the three components of SES were not in collinearity.

## Discussion

In this community-based study conducted in Chinese adults in Jiaxing, a typical city undergoing a rapid urbanization in China, we find that the intensity of overall PA in this population was 165 MET-hours / week, much lower than the average national level in 2010. The sedentary time in this population was only 18 hours/week, also about 2 hours/week less than the national level estimated by Ng, *et al* [32]. Our results suggest an overall insufficiency in PA, possibly due to the lack of high-intensity activities, in our population. More importantly, in this population both PA types and intensity were significantly different by SES: the middle SES groups had higher intensity of occupational activities, the lowest SES groups engaged in more household activities, whereas those with highest SES were more likely to exercise but had more sedentary behaviors. All the three components of SES, education attainment, income and occupation, contributed to socioeconomic disparities in PA.

It has been suggested that leisure-time activities and occupational activities were the main PA types in European countries [10]. In our population, however, energy expenditure in occupational activities was found to be the major contributor to the intensity of overall PA (82%), and was most closely associated with SES and its components, suggesting the central role of occupational activities in examining SES inequalities in PA. Possibly due to the high unemployment rate (27.9%) in the lowest SES class and the more sedentary occupational activities engaged in the highest SES stratum, the two groups had much lower occupational PA levels than the two middle SES groups. Unlike the lowest SES class who greatly increased their total intensity of PA by engaging in household activities, the highest SES class was not offset for their lower intensity in occupational PA with any other types of activities. Although the Upper class had distinctly higher energy expenditure in exercise than did other SES classes, only 23.6% of them engaged in exercise, with an average volume of only 0.9 MET-hours / week. Therefore, the Upper SES subjects become the most disadvantaged class in the view of PA intensity, and possibly exposed to a greater risk of NCDs [7]. Furthermore, considering that occupational activities have been decreasing with the continually rapid urbanization in both urban and rural China in recent decades [20], it is expected that the middle classes, the groups having the highest occupational activity level but lacking leisure time exercise, will join the least active class in the near future. Apparently, the rapid decrease in occupational activities and housework and lack of exercise in this population may result in a high prevalence of NCDs and a large disease burden for the local government. Our results implicate an important and urgent promotion in leisure-time activities in this population.

SES influences PA behaviors by several approaches, including self-efficacy and social support [33]. Usually, lower education may result in one’s unawareness of positive health consequences of PA, and therefore decrease their self-efficacy to exercise [34]; lower SES people are less likely to afford PA equipment or sports facilities, and are also less likely to participate in free activities like walking and running due to some social factors such as competing pressure or childcare [35]. Moreover, physical environment has an extremely important influence on opportunities to be physically active [20,36]. People from lower socioeconomic stratum usually have poorer access to physical environments such as parks, gardens, stadiums and other facilities that enable individuals to take exercise. Some cultural and intrapersonal factors may also play an indirect role in SES disparities in PA [37]. In most previous studies conducted in developed countries, lack of leisure-time PA in lower SES groups due to their limited access to sports facilities has been the main concern in socioeconomic inequalities [10,11,38,39].

In this study, of the three components of SES, occupation and educational attainment but not income level was significantly associated with intensity of exercise. The results were somewhat inconsistent with most previous studies in which income was one of determinants of leisure-time PA [11,13,18,40,41]. Our results indicate that affordability may not be the main factor that influences the engagement of leisure-time activities in this population. It seems that low self-efficacy of exercise existed not only in low SES class but also in those highly educated or highly paid. Therefore, interventions and policies should be invested to promote leisure-time activities among all adults. In other words, health promotion and policy efforts should be elicited to improve incentives to take exercise in the whole population.

The main strength of this study is our first attempt to adopt SEI score to assess socioeconomic status comprehensively, which makes it possible to evaluate the effect of SES comprehensively. Moreover, the comprehensive measurement of PA enables us to examine a broad range of PA at work, at home, during commuting and leisure time. These measurements, combined with additional measurement of sedentary time, provided us a panorama of daily behaviors that represent the popular modern lifestyles in a population experiencing a nutrition transition.

Several limitations of this study should be mentioned. First, the cross-sectional design makes it difficult to reveal a causal relationship between SES and PA. Second, our results were derived from a community-based survey conducted in Jiaxing, a small city covering only 4.50 million residents, which may be just a window of China in transition. Third, the data on PA and socioeconomic position were self-reported, and thus may be subject to recall bias. Finally, we did not collect information on environmental factors such as local resources, supply of sports facilities and intrapersonal configuration of individuals, and thus could not evaluate the effects of social support and self-efficacy on PA behaviors in our population.

In summary, this study shows a comprehensive association of SES with intensity of different PA types in Chinese adults, and outlines the unique socioeconomic inequalities in PA in a population experiencing the rapid urbanization.

## Supporting Information

S1 Table Socioeconomic index score for Chinese urban residents, 2010 (DOCX)
