# Supplementary material for: Socioeconomic Status and Physical Activity in Chinese Adults: A Report from a Community-Based Survey in Jiaxing, China
Source: PLoS One. 2015 Jul 15;10(7):e0132918. doi: 10.1371/journal.pone.0132918 (PMC4503452; doi:10.1371/journal.pone.0132918)
Supplement: S1 Table — (DOCX) [file pone.0132918.s001.docx]

| S1 Table. Socioeconomic index score for Chinese urban residents, 2010 | | |
| --- | --- | --- |
| Socioeconomic components | Classification | Score |
| Educational level | | |
|  | College or above | 6 ^a^ |
|  | High school | 4 |
|  | Middle school | 3 |
|  | Elementary school | 2 |
|  | Illiterate or semiliterate | 1 |
| Occupation | | |
|  | Administration staff | 6 |
|  | Professionals | 5 |
|  | Clerk | 4 |
|  | Service Personnel | 3 |
|  | Manual worker | 2 |
|  | Unemployed | 1 |
| Annual income per capita (USD) | | |
|  | ≥ 11,804 | 7 |
|  | 7,870~11,803 | 6 |
|  | 5,903~7,869 | 5 |
|  | 3,935~5,902 | 4 |
|  | 1,968~3,934 | 3 |
|  | 985~1,967 | 2 |
|  | ≤ 984 | 1 |

^a^ According to Li’s criteria, educational attainment of college or above includes graduating from junior college (score 5), senior college (score 6) and graduate school (score 7). Due to lack of the details, score of 6 was used as an average level for the group.
